# A new species in the genus *Amphipteryx* Selys, 1853 (Odonata, Amphipterygidae) from Pico Bonito National Park, Honduras

**DOI:** 10.3897/zookeys.408.7174

**Published:** 2014-05-13

**Authors:** Merlijn Jocque, Ivany Argueta

**Affiliations:** 1 Jessica Ware Lab, Rutgers, the State University of New Jersey, 195 University Ave, Newark, NJ, 07102, USA; 2 Royal Belgian Institute of Natural Sciences (RBINS), Vautierstraat 29, 1000 Brussels, Belgium; 3 Biodiversity Inventory for Conservation (BINCO), Rijmenamsesteenweg 189, Haacht, Belgium; 4 Instituto Nacional de Conservación y Desarrollo Forestal, Áreas Protegidas y Vida Silvestre (ICF), Oficina Regional Forestal del Atlántico, Col. Palmira, La Ceiba, Honduras.

**Keywords:** Zygoptera, cloud forest

## Abstract

The Mesoamerican damselfly genus *Amphipteryx* includes four species: *Amphipteryx agrioides* (Mexico), *A. chiapensis* (Mexico), *A. meridionalis* (Honduras) and *A. nataliae* (Verapaz, Guatemala). We describe a fifth species, *Amphipteryx jaroli*, from the cloud forest in Pico Bonito National park, Honduras. Additionally we include an up to date key of all species in the genus for both sexes.

## Abstract (es)

El género Mesoamericano del zigóptero *Amphipteryx* incluye cuatro especies: *Amphipteryx agrioides*, (Mexico), *A. chiapensis* (Mexico), *A. meridionalis* (Honduras) y *A. nataliae* (Verapaz, Guatemala). Se describe una quinta especie, *Amphyterix jaroli*, localizada en ul bosque nublado del Parque Nacional Pico Bonito, Honduras. Adicionalmente se incluye una clave actualizada de todas las especies del género, tanto para machos como hembras.

## Introduction

Central American cloud forests habitats are critically endangered ecosystems disappearing rapidly (Solorzano et al. 2003). While an estimated 80% of original lowland forest vegetation cover has already been lost or modified (Brooks et al. 2002), these hilltop and ridge forests have remained untouched for a long time, mostly due to difficult access. The growing pressure on remaining natural resources associated with increasing population now is also affecting cloud forests. The difficult access, once a protective trait, now works against an efficient conservation as it hampers data collection. Relatively little is known on the biodiversity in these habitats, but cloud forests are characterised by a high diversity and high endemicity. A recent overview of the 100 most irreplaceable places for biodiversity in the world (Saout et al. 2013) included the cloud forest in Pico Bonito National Park.

During a biodiversity survey of cloud forest in La Montaña de Corazal (Pico Bonito National Park) we discovered a new species of *Amphipteryx*. The genus *Amphipteryx* belongs to the monotypic family Amphipterygidae (Dijkstra et al. 2013). The two latest overviews of the species in this genus (González-Soriano and von Ellenrieder 2009; González-Soriano 2010) present illustrations and a key to all known species. The species from Pico Bonito NP has a close resemblance to *Amphipteryx meridionalis*, the only other *Amphipteryx* currently recorded from Honduras. The new species differs by the bilobed lamellate processes on the prothorax. Here we describe this new species of *Amphipteryx* and update the keys for the separation of all species within the genus.

## Materials and methods

Type material is deposited in the Royal Belgian Institute for Natural Sciences in Brussels Belgium (I.R.Sc.N.B.). Nomenclature follows Westfall and May (2006) for body morphology. All measurements are in mm; total length and length of abdomen for up to 10 specimens of each sex include cerci; means (in parenthesis) are given for more than two specimens. All drawings were made with the aid of a camera lucida coupled to a Nikon SMZ1500 stereoscope and are not to scale. Map represents distribution records from collections, and was created using QGIS 2.0. Abbreviations for structures used throughout the text are as follows: Fw: forewing; Hw: hindwing; pt: pterostigma; Ax: antenodal crossveins; Px: postnodal crossveins; S1–10: abdominal segments 1 to 10.

## Results

### Amphipteryx

Selys, 1853

http://species-id.net/wiki/Amphipteryx

Amphipteryx Selys 1853: 66.

#### Type species.

*Amphipteryx agrioides* Selys 1853, by original designation.

**Figure 1.**
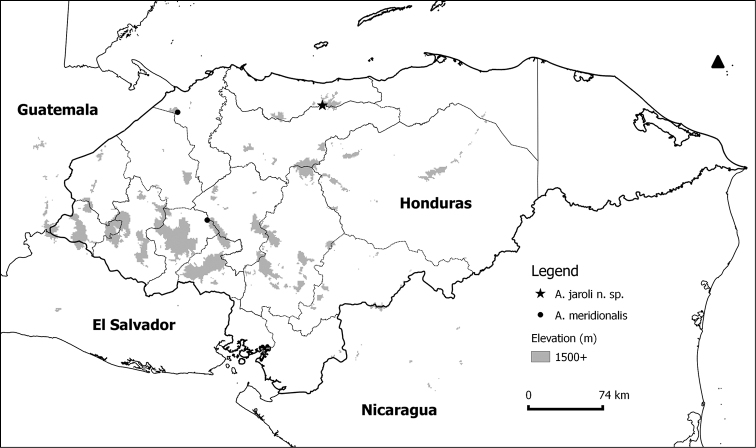
Occurrence records of the two species of *Amphipteryx* found in Honduras. Arrow indicates the North.

#### Other species included.

*Amphipteryx chiapensis* González–Soriano 2010, *Amphipteryx meridionalis* González-Soriano 2010, *Amphipteryx nataliae* González-Soriano 2010.

#### General.

*Amphipteryx* is the only genus in Amphipterygidae (Dijkstra et al. 2013). *Rimanella*, with its monotypic species *Rimanella arcana* (Needham 1933), was formally included in Amphipterygidae but it was recently placed in its own monotypic family Rimanellidae (Dijkstra et al. 2013).

#### Distribution.

*Amphipteryx* occurs at small mountain streams from Hidalgo Oaxaca, Puebla and Veracruz States, Mexico east into Guatemala and Honduras.

#### Biology.

Adults perch with wings closed on vegetation overhanging water near seepages and small streams (González-Soriano 1991); larvae live in rough gravel and rapid-flow areas of small shallow creeks, and among leaf litter at lips of small waterfalls (Novelo-Gutiérrez 1995). With one exception (below) all Mexican populations are associated with tropical wet forests (i. e. cloud or tropical rain forests) and the same apparently is true for other Central American populations.

#### Key to males of *Amphipteryx*

This key is a modification of the one from González-Soriano (2010) and reference should be made to illustrations for other species in that paper.

**Table:** 

1	Hind lobe of prothorax evenly curved with small disjunct lateral lobes forming small rounded lobes whose medial margins are bent anteriorly; cercus in dorsal view curved medially and armed with a blunt quadrate lobe along medial 0.40; paraproct slender, slightly surpassing cercus and with medially curved tip terminating in a single tooth; Hidalgo and Oaxaca States, Mexico	*Amphipteryx agrioides*
1’	Hind lobe of prothorax with erect digit-like lateral lobes, or with middle lobe bent cephalad or posteriorly (Fig. 2D); cercus in dorsal view linear and lacking a blunt quadrate lobe along medial 0.40, paraproct dorso-ventrally flattened, as long as or considerably shorter than cercus and terminating in an angular to quadrate tip; Chiapas State, Mexico, Honduras and Guatemala	2
2(1’)	Hind lobe of prothorax continuous; raised lateral lobes vertical, semicircular, and continuous with smaller irregular shaped middle lobe; Honduras	3
2’	Hind lobe of prothorax with erect digit-like lateral lobes; Chiapas State, Mexico, and Guatemala	4
3(2)	Scalariform tooth on cercus in mediodorsal view anteapical, medial margin of cercus gently concave; middle lobe of hind lobe of prothorax bilobed or with a v-shaped incision medially, bent cephalad, this structure as prominent as lateral lobes; Comayagua and Cortés Departments, Honduras	*Amphipteryx meridionalis*
3’	Scalariform tooth on cercus in mediodorsal view at medial half, medial margin of cercus with ventro-medial lobe at basal third (Fig. 2A); middle lobe of hind lobe of prothorax entire, bent posteriorly and much smaller than erect wing-like lateral lobes (Fig. 2D); Atlantida Department, Honduras	*Amphipteryx jaroli*
4(2’)	Paraproct as long as cercus and terminating in an angular tip; erect lateral lobes of prothorax as long as interval between them; Guatemala	*Amphipteryx nataliae*
4’	Paraproct considerably shorter than cercus and terminating in a quadrate tip; erect lateral lobes of prothorax much shorter than interval between them; Chiapas State, Mexico	*Amphipteryx chiapensis*

#### Key to females of *Amphipteryx* (female of *Amphipteryx chiapensis* unknown)

**Table:** 

1	Hind lobe of prothorax evenly curved with lateral lobes forming small angulate lobes; Hidalgo and Oaxaca States, Mexico	*Amphipteryx agrioides*
1’	Hind lobe of prothorax with erect digit-like lateral lobes, or with middle lobe bent cephalad or posteriorly (Fig. 2E); Chiapas State, Mexico, Honduras and Guatemala	2
2(1’)	Hind lobe of prothorax with a pair of small, isolated, erect digit-like lateral lobes converging posteriorly; Guatemala	*Amphipteryx nataliae*
2’	Hind lobe of prothorax with erect lateral lobes continuous with middle lobe; Honduras	3
3(2’)	Middle lobe of hind lobe of prothorax bilobed or with a v-shaped incision medially, bent cephalad, this structure as prominent as lateral lobes; Comayagua and Cortés Departments, Honduras	*Amphipteryx meridionalis*
3’	Middle lobe of hind lobe of prothorax entire, bent dorso-posteriorly and subequal in height to erect wing-like lateral lobes (Fig. 2E); Atlantida Department, Honduras	*Amphipteryx jaroli*

### Species description

#### Amphipteryx jaroli sp. n.

http://zoobank.org/DAE4DF4E-0E95-4A33-BA0C-5E02CE4EF531

http://species-id.net/wiki/Amphipteryx_jaroli

Figs 2A–E , 3A–E

##### Etymology.

Named *jaroli* (noun in the genitive case), after our friend and guide through the cloud forest on our first expedition in Pico Bonito National Park (2012); Jarol Estrada. Jarol collected the first specimen of this species.

##### Type material.

Total: 9 males (32.597/1-10, Coll. I.R.Sc.N.B.) and 1 female (32.597/10). Holotype: male (32.597/1), Honduras, Pico Bonito National Park., Montaña de Corazal, cloud forest, north of the small village Los Horcones in Northern Honduras. Basecamp was at 1640m (N15.556, W86.918), all collections were made in the vicinity from this location.

##### Description.

Holotype dimensions: Fw 39,0 mm; Hw 36,0 mm; abdomen 46,0 mm; total length 51,0 mm. Head of male holotype with basal part of labium light cyan blue (Fig. 3D), labial palp and apex of mentum black, labrum and gena light cyan blue, mandible black except for well-defined pale yellow spot at base, large spot confluent with the genae on either side of antefrons, light cyan blue (Fig. 3A, C–D). Clypeus and midline of antefrons black, and most of the dorsal and posterior surfaces of the head, black, lateral area of antefrons light cyan blue; anterior portion of epicranium to epicranial furrow shiny black except for a small elongated yellow patch extending antero-laterally from each lateral ocellus (Fig. 3B); posterior portion of epicranium including postocular lobes, occipital bar, and rear of head matte black.

**Figure 2.**
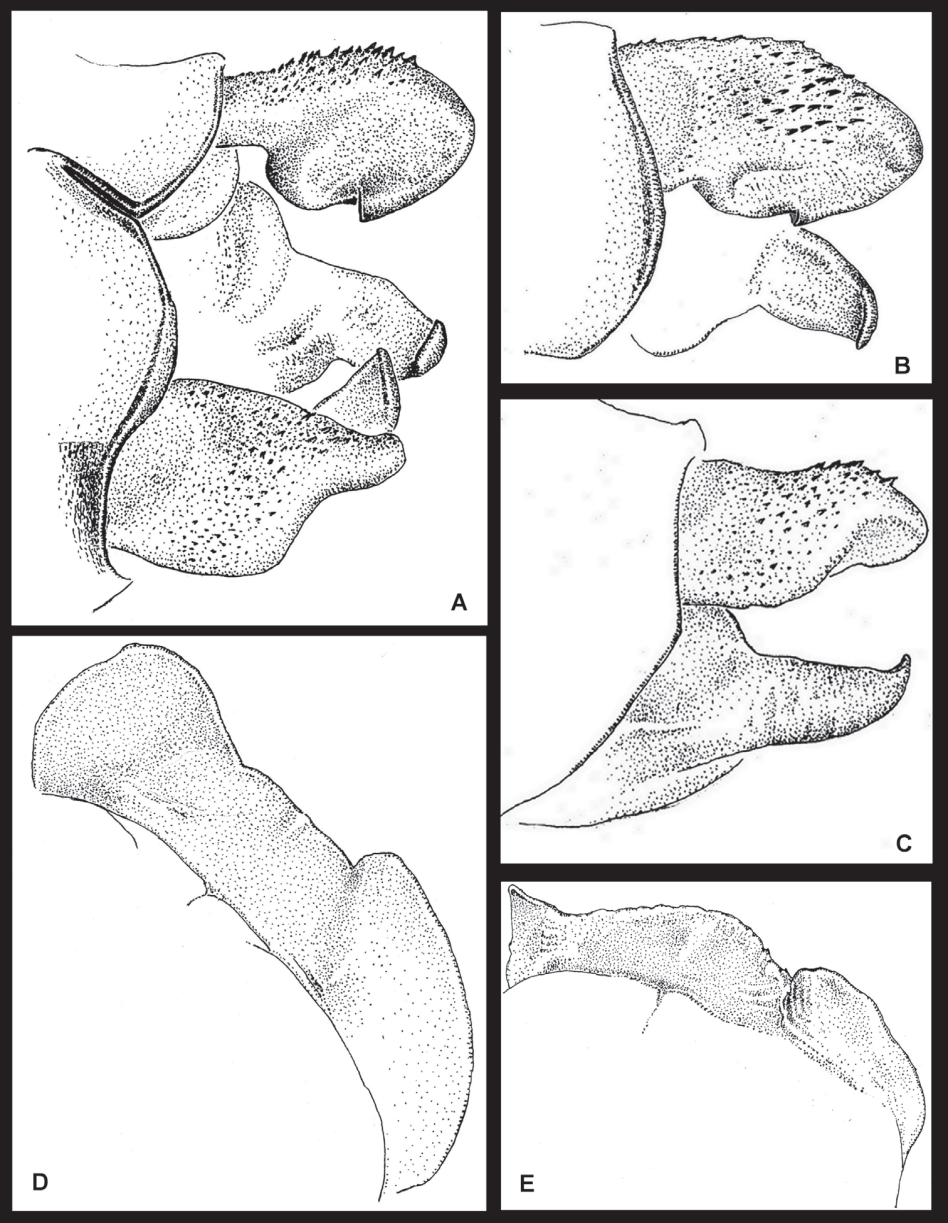
*Amphipteryx jaroli* (**A–E**), S10 holotype male dorso-lateral (**A**), ventro-lateral (**B**) and lateral (**C**) view. Holotype male posterior lobe of prothorax (**D**) and female posterior lobe of prothorax (**E**).

**Figure 3.**
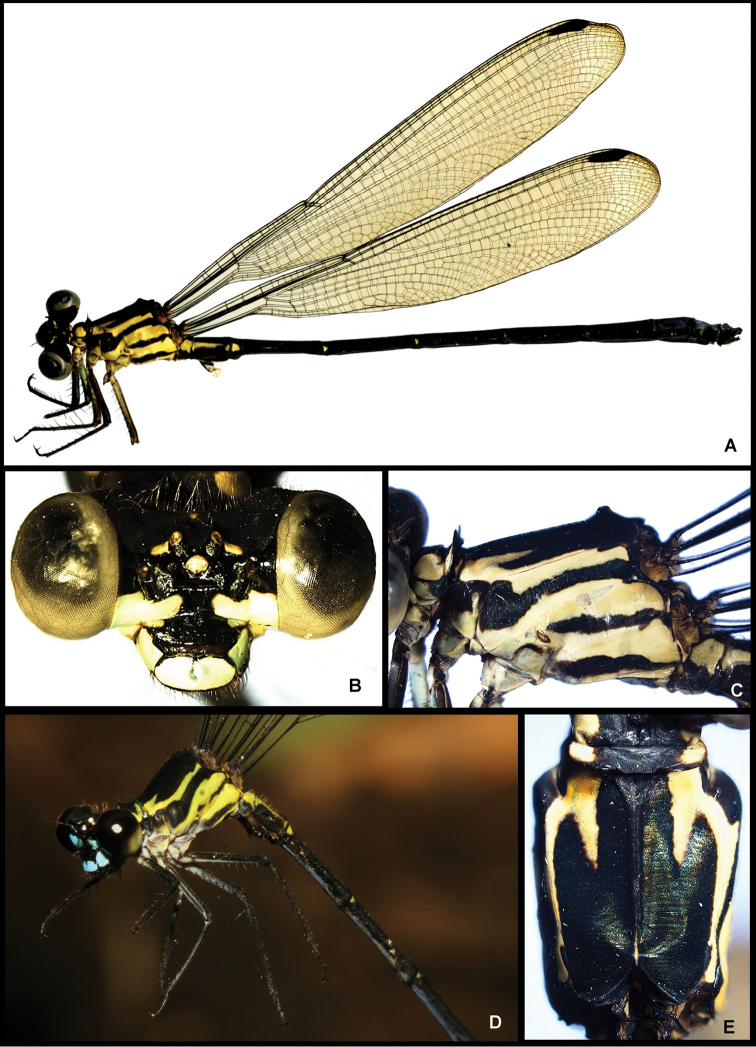
Images of *Amphipteryx jaroli* (**A–E**), whole male holotype dead and preserved (**A**), frontal view head (**B**), lateral pterothorax (**C**), life male in the field (**D**) and dorsal pterothorax (**E**).

Prothorax black except for pale yellow anterior lobe and large lateral spot on either side of median lobe and below ventral margin of propleural suture (Fig. 3A, C–D); hind lobe (Figs 2D, 3A, C) with paired dorsal, upright, lamellate processes, which, in lateral view (Fig. 3C) are thin, and strongly erect, each anteriorly with pale yellow patch on distal half; distance between these lobes about twice the height of each lobe, middle lobe decumbent dorso-posteriorly and not as high as lateral lobes. Synthorax yellow green (Fig. 3A, C–D) with a broad mid-dorsal black stripe confluent with abbreviated broad black antehumeral stripe at upper half, its ventral portion acuminate, and not reaching mesinfraepisternum; this last with anterior half black, posterior half pale yellow green; side of thorax yellow green with three narrow lateral black stripes; one on middle of mesepimeron with its ventral portion expanding to mesinfraepisternum, its upper end along margin of antealar crest and meeting second (interpleural) stripe at upper posterior margin of metepimeron and ending antero-ventrally above metastigma; third thoracic stripe extending full length of posterior margin of metepimeron; venter of thorax pale. Coxae pale yellow washed with darker brown ventrally; legs blackish with bases and the inner, surfaces of the femora yellowish; armature and claws black. Wings hyaline; Ax Fw 8:9; Hw 8:8; Px Fw 31:28; Hw 23:27.

Abdomen black (Fig. 3A) with following parts yellow green: a narrow mid-dorsal line on S2–5, dorso-lateral spot on S1, a narrow dorso-lateral line on S2, a decreasingly smaller latero-basal spot on S3, S4 and S5, dorsum of S7–10 bright light blue dorsally (faded to brown due to postmortem affects), a thin mid-dorsal black line on S10, its postero-dorsal margin with a narrow median notch half as long as segment. Genital ligula with two dorsal and two ventral lobes, semi-hyaline with light brown patch at the base of the ventral lobes; semi-hyaline dorsal lobes about twice as long as ventral lobes, ending in elongate spatula shaped rounded tip; semi-hyaline ventral lobes ending in rounded tip. Cercus black, subequal to S10, robust, spinulose dorso-externally, in dorsal view fusiform with apex rounded, medial side with a well-developed scalariform tooth approximately halfway between apex and base (Fig. 2A, C), medial surface from tooth to base with a large robust ventro-medially directed lobe (Fig. 2A); medial surface from tooth to apex smooth, slightly convex; in lateral view cercus linear, with ventral surface dorsally arched exposing inner margin of tip of cercus (Fig. 2A). Paraproct subequal to cercus, its tip in lateral view directed dorsally (Fig. 2B), base in dorsal view inflated, paraproct tapering down, apex with quadrate tip pointing medio-dorsally (Fig. 2A, C).

##### Variation in paratypes.

Males differ in extent of black on body and in shape of pronotal lobes. The narrow mesepimeral stripe in some is complete and connects with black ventrally on mesinfraepisternum, interpleural stripe may be broken into elongate spots and third thoracic stripe may be reduced. Normally the pronotal lobes are pale, but the size of this patch varies in some specimens, height and morphology of the pronotal lobes is also variable. In some animals the normally erect lobes gently dip anteriorly when viewed laterally. In one male, wings were slightly infumed.

Female similar to male but black markings on head, pro- and especially on synthorax reduced. Mid-dorsal black stripe narrower than in male, occupying less than 0.25 of each mesepisternum, asymmetrical hourglass pattern, with the narrowest part anterior, steadily widening posteriorly, ventral acuminate portion isolated, only attached to mid dorsal stripe through a narrow black line at the edge of the pterothorax, in dorsal view an acuminate fish hook shape, extends less than half length of mesepisternum. Pronotal lobes smaller and less pronounced compared to male.

##### Dimensions.

Males (*n* = 25, including holotype; means in parentheses): Hw 34.0–37.5 mm (35.9±1.0 mm); abdomen 39.5–43.0 mm (41.5±0.9 mm); total length 50.0–54.0 mm (52.1±1.0 mm). Females (*n* = 2): Hw 38.0 mm; abdomen 37.5 mm; total length 50.5 mm.

##### Distribution.

Currently only known from Pico Bonito National Park, Honduras (Fig. 1). *Amphipteryx jaroli* was collected from three neighboring river catchments, all within a narrow elevational range (1611 to 1673 masl).

##### Diagnosis.

The structure of the pronotal lobes in lateral view, with the two large, straight lobes, separates this species from other species in the genus. Additionally, the male is separable based on the position of the well-developed scalariform tooth on the internal side of the cercus approximately halfway between apex and base. *Amphipteryx chiapensis*, *Amphipteryx meridionalis* and *Amphipteryx nataliae* have a well-developed scalariform anteapical tooth, *Amphipteryx agrioides* has the scalariform tooth positioned more basally, but still closest to the apex (González-Soriano and von Ellenrieder 2009).

## Discussion

The narrow elevational distribution of *Amphipteryx jaroli* is remarkable. The expedition departed from Los Horcones, a small village at an elevation of 289m, crossing the forest on a three day hike to a provisionary basecamp at an elevation of 1640masl. From basecamp daily excursions in all directions were completed and dragonflies collected. Despite the substantial altitudinal range crossed, this species was only collected from a narrow elevational range. Other *Amphipteryx* species are also cloud forest species as a rule occurring at mid-height elevation around medium sized rocky mountain rivers. A single *Amphipteryx agrioides* was collected at 36m, but this seems to be an exception. *Amphipteryx agrioides* has been found as low as 650m in Pueblo State, Mexico.

## Supplementary Material

XML Treatment for Amphipteryx

XML Treatment for Amphipteryx jaroli
